# Genome-Wide Comparative Analysis of SRCR Gene Superfamily in Invertebrates Reveals Massive and Independent Gene Expansions in the Sponge and Sea Urchin

**DOI:** 10.3390/ijms25031515

**Published:** 2024-01-26

**Authors:** Zhangjie Peng, Wei Zhang, Hailun Fu, Yuzhu Li, Chunyu Zhang, Jie Li, Jiulin Chan, Linlin Zhang

**Affiliations:** 1 College of Life Sciences, School of Marine Science and Engineering, Qingdao Agricultural University, Qingdao 266109, China; zhangjiepeng996@163.com (Z.P.); akumaev@163.com (H.F.); 17852427120@163.com (Y.L.); 17852021312@163.com (C.Z.); 2 CAS and Shandong Province Key Laboratory of Experimental Marine Biology, Center of Deep-Sea Research, Institute of Oceanology, Chinese Academy of Sciences, Qingdao 266071, China; 3 College of Marine Science, University of Chinese Academy of Sciences, Beijing 100049, China; 4 Laboratory for Marine Biology and Biotechnology, Qingdao National Laboratory for Marine Science and Technology, Qingdao 266071, China; 5 Key Laboratory of Breeding Biotechnology and Sustainable Aquaculture, Chinese Academy of Sciences, Qingdao 266071, China

**Keywords:** SRCR gene family, innate immunity, gene duplication, independent evolution, motif analysis

## Abstract

Without general adaptative immunity, invertebrates evolved a vast number of heterogeneous non-self recognition strategies. One of those well-known adaptations is the expansion of the immune receptor gene superfamily coding for scavenger receptor cysteine-rich domain containing proteins (SRCR) in a few invertebrates. Here, we investigated the evolutionary history of the SRCR gene superfamily (SRCR-SF) across 29 metazoan species with an emphasis on invertebrates. We analyzed their domain architectures, genome locations and phylogenetic distribution. Our analysis shows extensive genome-wide duplications of the SRCR-SFs in *Amphimedon queenslandica* and *Strongylocentrotus purpuratus*. Further molecular evolution study reveals various patterns of conserved cysteines in the sponge and sea urchin SRCR-SFs, indicating independent and convergent evolution of SRCR-SF expansion during invertebrate evolution. In the case of the sponge SRCR-SFs, a novel motif with seven conserved cysteines was identified. Exon–intron structure analysis suggests the rapid evolution of SRCR-SFs during gene duplications in both the sponge and the sea urchin. Our findings across nine representative metazoans also underscore a heightened expression of SRCR-SFs in immune-related tissues, notably the digestive glands. This observation indicates the potential role of SRCR-SFs in reinforcing distinct immune functions in these invertebrates. Collectively, our results reveal that gene duplication, motif structure variation, and exon–intron divergence might lead to the convergent evolution of SRCR-SF expansions in the genomes of the sponge and sea urchin. Our study also suggests that the utilization of SRCR-SF receptor duplication may be a general and basal strategy to increase immune diversity and tissue specificity for the invertebrates.

## 1. Introduction

Immunity refers to the function of the body’s immune system to recognize self and non-self substances and eliminate antigenic foreign substances through immune responses to maintain physiological balance [1,2]. The immune system consists of innate immunity and acquired immunity [3], which are composed of immune organs, immune cells, and immune active substances, and have various functions such as immune surveillance, defense, and regulation [4]. In the kingdom Animalia, more than 95% are invertebrates [5,6]. It is generally believed that invertebrates lack acquired immunity and depend solely on innate immunity for pathogen resistance, encompassing the entire process from pathogen recognition to elimination [7]. Pathogen-associated molecular patterns (PAMPs) are a class of conserved molecular structures found on the surface of pathogens, including bacteria, viruses, and fungi [8]. Pattern recognition receptors (PRRs) are non-clonal recognition molecules distributed on the surface of natural immune cells that can recognize PAMPs [9]. The PRR family includes Toll-like receptors (*TLRs*), scavenger receptors with cysteine-rich domains (*SRCRs*), C-type lectin receptors (*CLRs*), and nucleotide-binding oligomerization domain-like receptors (*NLRs*), among others [10,11]. As an important receptor family in the innate immune system, PRRs recognize and interact with PAMPs on the surface of pathogens [12], which is critical for initiating innate immune responses [13]. Once these patterns are recognized, PRRs trigger a series of cell signaling events, activate immune responses, and help clear pathogens from the body.

The scavenger receptor cysteine-rich gene superfamily (SRCR gene superfamily, SRCR-SF) is a receptor family that is rich in cysteine residues and was first proposed in the 1990s [14]. The structure of SRCR-SFs is diverse, but most contain an N-terminal signal peptide, one or multiple SRCR domains, a transmembrane domain, and a C-terminal cytoplasmic tail [15]. The number and arrangement of these domains differ among various subfamilies of SRCR-SFs. The broad criteria have led to a lack of detailed classification of SRCR-SF members, with differentiation being based solely on the type of SRCR domain. SRCR domains are divided into Group A and Group B based on the number and position of conserved cysteine residues [16,17], with Group A having six cysteine residues that form three pairs of disulfide bonds, and Group B having eight cysteine residues that form four pairs of disulfide bonds [18]. For instance, CD5 (cluster of differentiation 5) [19], CD6 (cluster of differentiation 6) [20], and DMBT1 (deleted in malignant brain tumor 1) [21] belong to Group B, whereas MARCO (macrophage receptor with collagenous structure) [22], Mac2BP (Mac-2 binding protein) [23], and LOX (lysyl oxidase) family (LOX-like 2, LOX-like 3, and LOX-like 4) [24] belong to Group A. Until now, research on SRCR-SFs within Group B has primarily focused on vertebrates, with only one reported instance in the invertebrate *Geodia cydonium* [25]. Furthermore, no SRCR-SF has been found to have both Group A and Group B SRCR domains [26]. The SRCR-SFs participate in the recognition and binding of various ligands, including lipids, proteins, carbohydrates, and other molecules [27]. The diverse functions of SRCR-SFs as a pattern recognition receptor have all been extensively studied, including pathogen recognition, modulation of the immune response, maintenance of epithelial homeostasis, involvement in stem cell biology, and contribution to tumor development [28,29]. An expanded superfamily of 218 SRCR-SF models in the sea urchin [30] was first reported. Subsequently, the expansion of SRCR-SFs was also identified in the Pacific oyster (*Crassostrea gigas*) [31] and the amphioxus (*Branchiostoma floridae*) [32]. Examination of SRCR-SFs in the scallop *Chlamys farreri* revealed their involvement in immune responses, not only to bacterial invasion but also to fungal invasion [33]. These findings underscore the potential utilization of SRCR-SF receptors as a general strategy in invertebrate immune recognition [18,28] and suggest the possibility of predicting features of an ancestral bilaterian pattern. However, a large-scale overview of the SRCR-SF’s macroevolution pattern is still absent.

In this study, we delved into the immune diversity and evolutionary history of SRCR-SFs across metazoans, with a particular focus on invertebrates. Our analysis encompassed the identification of 1747 members of SRCR-SFs in 29 metazoans. Notably, we observed a significant expansion in gene numbers within the genomes of the sponge and the sea urchin. Separately, our investigation extended to analyzing their domain architecture and phylogeny, revealing a remarkably diverse composition of domains within SRCR-SFs. We meticulously examined genomic locations, exon–intron structures, and motif arrangements to elucidate the molecular mechanisms driving this extensive expansion. Our genome-wide comparative analysis of SRCR-SFs highlighted an independent evolution of gene expansion specific to the sponge and sea urchin. This finding underscores the likelihood that the dynamic duplication of SRCR-SF genes represents a widespread strategy for immune recognition in invertebrates, potentially serving as a foundational mechanism for immune processes in metazoans.

## 2. Results

### 2.1. Identification of SRCR-SFs in Metazoans

**Figure 1:**
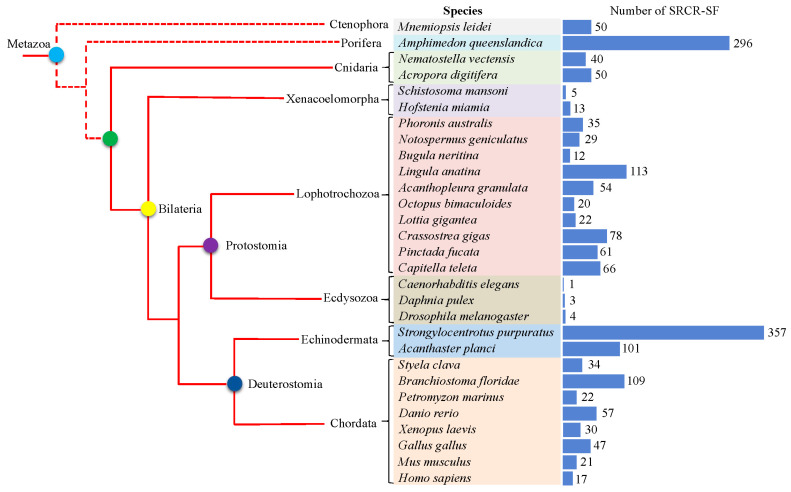
The distribution of members of the SRCR-SFs in 29 representative animal species across metazoans. The colors of species represent different phyla. The red cladogram on the left illustrates the phylogenetic relationships between species, with dashed lines indicating the disputed phylogenetic positions of ctenophores and sponges. The blue histogram on the right chart tallies the total number of identified SRCR-SFs for each species.

To study the evolution history of SRCR-SFs in metazoans, we combined sequence homology-based and signature domain predication tools to annotate SRCR-SFs in the genome of 29 representative species with different evolutionary positions. We annotated a total of 1747 SRCR-SF genes (Supplementary Table S7) [34,35,36,37,38,39] and found that SRCR-SFs are widely present in metazoans (Figure 1). A total of 296 SRCR-SFs were annotated in the genome of the sponge *Amphimedon queenslandica* of the phylum Porifera, suggesting the massive expansion of SRCR-SFs in the sponge. Besides the sponge, massive expansion of SRCR-SFs was also found in the Echinodermata *Strongylocentrotus purpuratus*, resulting in 357 SRCR-SF gene models. The expansions of SRCR-SFs were also found in the Chordata *Branchiostoma floridae*, the Echinodermata *Acanthaster planci*, and the Brachiopod *Lingula anatina*. The findings above indicate that employing the replication of SRCR receptors could be a pivotal strategy in augmenting immune diversity among metazoans [40,41]. Additionally, gene family constriction was also a general pattern during metazoan evolution [40,41]. For example, 5 and 13 SRCR-SFs were identified in the bilaterian basal clade *Schistosoma mansoni* and *Hofatenia miamia*. In the Ecdysozoa clade, no more than four SRCR-SF gene models were predicted across the three studied species. Notably, within the Lophotrochozoa clade, which comprises one of the most species-rich marine fauna [42], the diversity in the number of gene models encoding SRCR-SFs was evident. For instance, the Brachiopod *Lingula anatina* exhibited 113 identified SRCR-SFs, while the Bryozoa *Bugula neritina* had only 12 annotated SRCR-SFs. Furthermore, bivalves, in general, showcased a higher count of gene models compared to those in gastropods.

### 2.2. Domain Architectures and Genomic Distribution of SRCR-SFs

Canonical SRCRs have a typical SRCR domain that mediates protein–protein interactions and ligand binding [43]. In the 29 representative species, we observed that SRCR-SFs exhibit diverse and intricate domain compositions (Supplementary Table S6). Out of the 1747 SRCR-SFs identified, 53.66% encode proteins consisting solely of the SRCR domain (Supplementary Table S4). Among the 123 domains predicted within SRCR-SFs, EGF-like, CUB, LDL-A, and Collagen stand out as the top 5 domains (Table 1). A total of 39 (25.16%) EGF_CA, 22 (14.19%) cEGF, 17 (10.97%) FXa_inhibition, 14 (9.03%) PK_Tyr_Ser-Thr, and 13 (8.39%) Astacin were identified in the sponge genome (Supplementary Table S5), and these domains represent the highest frequency within the sponge SRCR-SFs. Comparatively, a total of 127 (31.59%) EGF-like, 38 (9.45%) Sushi repeat, 36 (8.96%) F5/8 type C, 36 (8.96%) WSC, and 35 (8.71%) CUB were identified in the sea urchin genome (Supplementary Table S5).

Previous studies have demonstrated that tandem duplication serves as a primary force driving gene replication [44]. Accordingly, we aimed to determine whether tandem duplication contributes to the massive duplication phenomenon during SRCR-SF evolution. We categorized all identified SRCR-SFs into tandem and scatter groups, revealing that among 29 surveyed species, 8 species exhibited over 50% of their SRCR-SFs categorized within tandem groups, while 14 species showed over 40% of their SRCR-SFs falling into tandem groups (Supplementary Table S3). Concurrently, we observed a substantial expansion of SRCR-SFs among representative species belonging to the same clade, predominantly characterized by tandem group (Supplementary Figure S3). Consequently, we propose that the amplification of SRCR-SFs may be attributed to multiple tandem duplication events.

### 2.3. Expression Profiles of Metazoan SRCR-SFs

**Figure 2:**
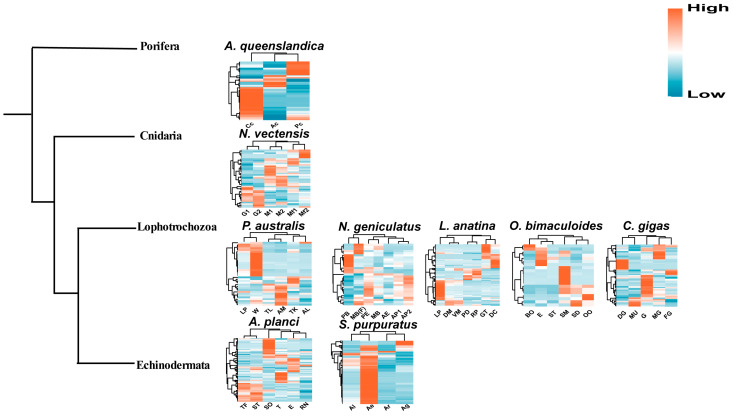
Transcriptome expression heatmaps of SRCRs of different tissues in nine representative metazoan species. *Amphimedon queenslandica:* archeocyte (Ac), choanocyte (Cc), pinacocyte (Pc; *Nematostella vectensis:* mesenterial filament 1 (Mf1), mesenterial filament 2 (Mf2), muscle 1 (M1), muscle 2 (M2), gonad 1 (G1), gonad 2 (G2); *Phoronis australis*: trunk with lophophore (TL), trunk (TK), lophophore (LP), ampulla (AM), actinotroch larva (AL), whole animal (W); *Notospermus geniculatus*: posterior end (PE), proboscis (PB), midbody with gut (MB), anterior part1 (AP1), anterior part2, posterior half without head (AP2), female midbody with gut (MB(F)), anterior end (AE); *Lingula anatina*: lophophore (LP), whole gut tissue (GT), digestive cecum (DC), dorsal mantle (DM), ventral mantle (VM), pedicle (PD), regenerated pedicle (RP); *Octopus bimaculoides*: sucker rims dissociated cells (SD), brain tissue from optic lobe (BO), eye (E), olfactory organ (OO), skin from mantle (SM), statocyst tissue (ST); *Crassostrea gigas*: digestive gland (DG), gill (G), male gonad (MG), female gonad (FG), muscle (MU); *Acanthaster planci*: stomach (SO), tube fee (TF), sensory tentacle (ST), eyes (E), radial nerve (RN), testes (T); *Strongylocentrotus purpuratus*: adult lantern (Al), adult guts (Ag), adult epidermis (Ae), adult radial nerve (Ar). Heatmap displays the expression level of SRCR genes.

Given the substantial expansions and diverse domain structures observed in metazoan SRCR-SFs, our investigation aimed to ascertain their functionality. To achieve this, we compiled all available lophotrochozoan tissue transcriptome data from the NCBI GEO database (up to October 2023). We conducted an analysis to determine the tissue-specific expression levels of SRCR-SFs in nine evolutionarily representative species (Figure 2, Supplementary Tables S7 and S8), encompassing the porifera *Amphimedon queenslandica*; the cnidaria *Nematostella vectensis;* the phoronidan *Phoronis austrailis*; the nemertean *Notospermus geniculatus;* the brachiopod *Lingula anatina*; the mollusks *Octopus bimaculoides* and *Crassostrea gigas*; and the echinodermata *Acanthaster planci* and *Strongylocentrotus purpuratus*. Among the 1069 SRCR-SFs studied in the nine species, clearly visible from the graphical representation is the heightened expression of SRCR-SFs within tissues linked to digestive glands and their associated functions across all species in the spectrum, which is notably evident in *Amphimedon queenslandica* and *Strongylocentrotus purpuratus.* This discovery indirectly demonstrates the expansion of SRCR-SFs within tissues associated with digestive function across various metazoans. Furthermore, it implies that the expansions of SRCR-SFs might confer advantageous effects on specific immune functions, such as mucosal immunity, within the digestive system of these metazoans. Collectively, these findings suggest that numerous SRCR-SFs exhibit high expression levels in immune-related tissues, specifically digestive glands, and potentially play a crucial role in innate immune recognition.

### 2.4. Extensive Expansion of SRCR-SFs in the Sponge

**Figure 3:**
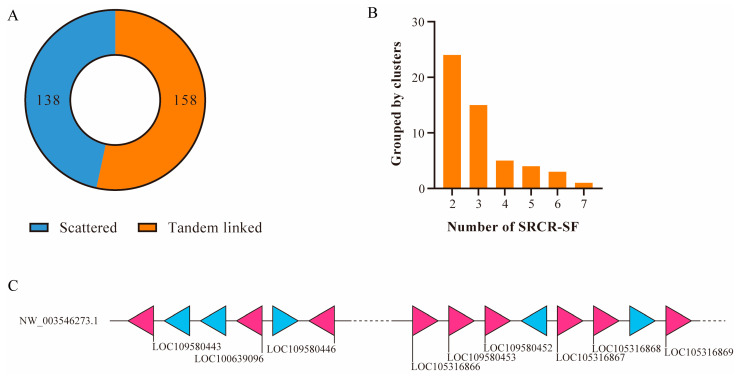
Genomic distribution analysis of sponge SRCR-SFs. (**A**) Proportions of clustered and individual members of sponge SRCR-SFs. Blue represents the 138 members of sponge SRCR-SFs scattered on the scaffolds; orange represents the 158 members of sponge SRCR-SFs linked in tandem array. (**B**) The histogram shows the tandem clusters containing 2, 3, 4, 5, 6, and 7 SRCR-SFs. (**C**) Gene distribution of the scaffold (NW_003546273.1) with the most SRCR-SFs in the sponge is shown. Pink represents SRCR-SFs, blue represents non-SRCR-SF genes. Numbers represent the quantity of SRCR domains encoded by a certain gene.

To understand the distinct expansion pattern of SRCR-SFs in metazoan evolution, we focused on investigating duplication mechanisms in the two species exhibiting notable SRCR-SF expansions. Initially, we scrutinized the genomic arrangements and exon–intron structures of SRCR-SFs in sponges. Our analysis revealed that 158 out of 296 (53.38%) SRCR-SFs were organized in tandem arrays (Figure 3A,B), indicating a potential origin of sponge SRCR-SFs through tandem gene duplication.

**Figure 4:**
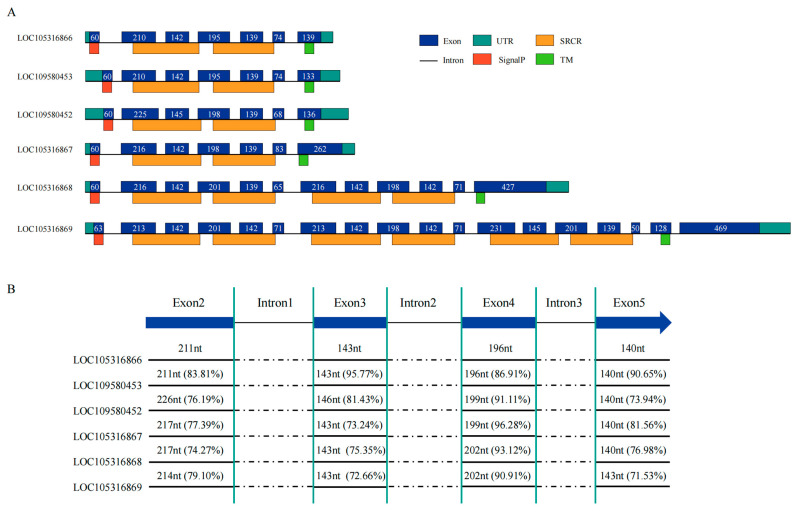
Gene structure analysis of sponge SRCR-SFs. (**A**) Gene structure of the tandem gene cluster with the largest number of sponge SRCR-SFs. Blue represents exon, green represents UTR, orange represents SRCR domains, black horizontal lines represent intron, light orange represents SignalP (signal peptide), light green represents TM, and numbers represent sequence lengths. (**B**) Sequence similarity of sponge SRCR-SFs. Blue represents exon, black horizontal lines represent intron.

Within these tandem clusters, NW_003546273.1 stood out, housing nine SRCR-SFs (Figure 3C) segregated into two subclusters by non-SRCR genes. Our focus narrowed to the longer subcluster encompassing six SRCR-SFs: LOC105316866, LOC109580453, LOC109580452, LOC105316867, LOC105316868, and LOC105316869. The initial four genes encoded similar domain architectures with two SRCR domains, while LOC105316868 and LOC105316869 were annotated with four and six SRCR domains, respectively (Figure 4A). Notably, all SRCR domains within these six SRCR-SFs displayed relative conservation in exon–intron architecture and sequence homology. For instance, LOC105316866 and LOC109580453 shared identical exon and intron structures, with six out of seven exons exhibiting matching sequence lengths. This suggests a potential recent tandem duplication event involving these genes (Figure 4B and Figure S1).

**Figure 5:**
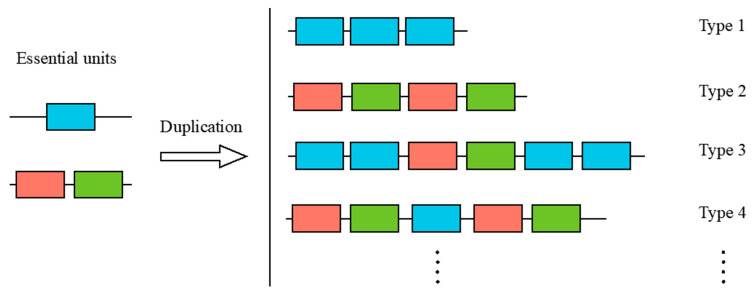
The replication units of SRCR-SFs. The predicted essential units include a single or two SRCR domains. Type 1 signifies that the SRCR gene is replicated from a single SRCR domain, while Type 2 signifies that the SRCR gene is replicated from two SRCR domains as a group. Type 3 and Type 4 signifies duplication with both Type 1 and Type 2 units.

Subsequently, we examined the replication unit within these tandem-linked SRCRs (Figure 5). In the NW_003546273.1 tandem SRCR-SFs, Type 1 and Type 2 SRCR-SFs were identified as duplicates, each containing one and two SRCR domains, respectively. Type 3 and Type 4 SRCR-SFs displayed duplication patterns involving both one and two SRCR domains. This led us to hypothesize that the varied ‘unit’ duplications might arise from disulfide bonds formed internally or interdomain within the SRCR domain.

### 2.5. Extensive Expansion of SRCR-SFs in Sea Urchin

**Figure 6:**
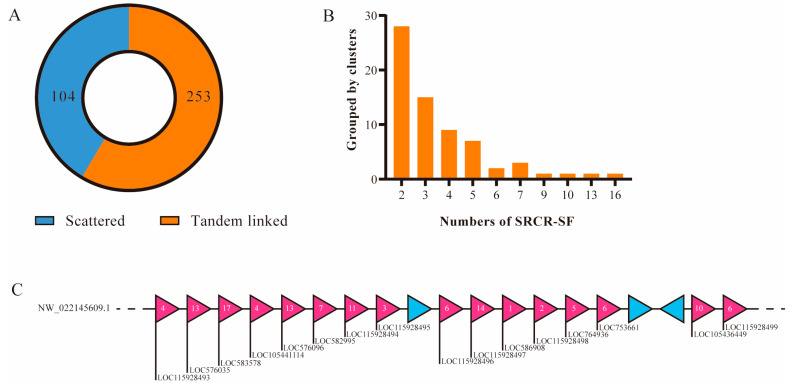
Gene distribution analysis of sea urchin SRCR-SFs. (**A**) The proportion of scattered and tandem linked SRCR-SFs in the sea urchin genome. Blue indicates that 104 SRCR-SFs are scattered on the scaffolds, while orange indicates that 253 SRCR-SFs are linked in tandem array. (**B**) A histogram showing the number of clusters containing 2, 3, 4, 5, 6, 7, 9, 10, 13 and 16 SRCR genes. (**C**) The gene distribution of SRCR-SFs on the scaffold (NW_022145609.1). Pink represents SRCR-SFs, blue represents non-SRCR-SF genes. Numbers represent the quantity of SRCR domains encoded by a certain gene.

To explore the extensive expansion of SRCR-SFs in sea urchin, we delved into deciphering the genetic basis of gene expansion within their genome. Contrasting with sponges, a higher proportion, 253 out of 359 (70.47%), SRCR-SFs in the sea urchin genome were organized into tandem clusters (Figure 6A). For instance, on scaffold NW_022145609.1, a tandem array accommodated 16 SRCR-SFs (Figure 6B), with 15 exclusively comprised of SRCR domains (Figure 6C).

**Figure 7:**
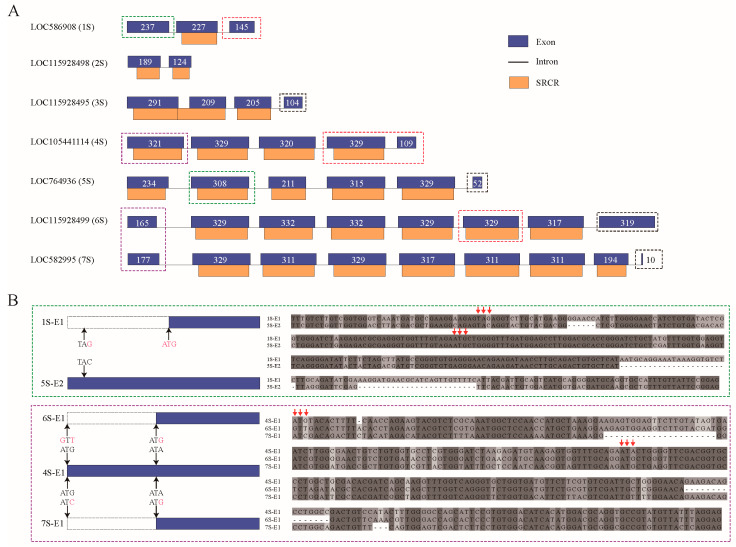
Gene structure analysis of sea urchin SRCR-SFs. (**A**) The gene structure of the SRCR-SFs in sea urchins is shown (gene cluster in Figure 6C). Blue represents exon, orange represents SRCR domain, black horizontal lines represent intron, and white numbers represent sequence length. (**B**) Analysis of special exon sequence alignment. Sequence alignment analysis was performed on the exons marked by the same color dashed box in (**A**). Red arrows mark the mutations.

In the genomic analysis, sea urchin SRCR-SFs showcased notably diverse exon–intron architectures. Examining these 16 SRCR-SFs revealed the presence of seven distinct exon–intron structure types. Interestingly, within sponge-linked SRCR-SFs, the majority encoded each SRCR domain across three exons. However, on scaffold NW_022145609.1, an exception emerged: all SRCR domains were encoded within a single exon, with the only deviation observed in the gene LOC115928495 (Figure 7A).

We aimed to ascertain the contribution of exon shuffling to SRCR-SF domain complexity [45]. This hypothesis was tested by identifying sibling paralogs with high sequence similarity based on the phylogenetic tree and comparing their exon–intron architectures. Widespread exon–intron structure divergence was evident within SRCR-SFs. In the sibling paralog pair LOC586908 and LOC764936, their corresponding exonic sequences exhibited significant alignment between LOC586908 exon 1 (1S-E1) and LOC764936 exon 2 (5S-E2), displaying 84% sequence similarity at the nucleotide level. An exon loss event was inferred in LOC586908, stemming from a stop codon mutation (TAC to TAG), resulting in the loss of an SRCR domain in the SRCR-SFs. A similar exon loss variation was also observed in the domain grafting of the sibling paralogs 6S-E1/4S-E1 and 7S-E1/4S-E1 (Figure 7B).

### 2.6. Comparative Analysis of SRCR-SF Distribution in the Sponge and Sea Urchin

The distribution and genetic basis of SRCR-SF expansion were investigated in sponge and sea urchin genomes to unravel distinct patterns of gene expansion. In the sponge, 53.38% of SRCR-SFs were organized in tandem arrays, suggesting a potential origin through tandem gene duplication. Within specific clusters, such as NW_003546273.1, six SRCR-SFs exhibited conserved exon–intron structures and domain architectures, indicating recent tandem duplication events. These duplications displayed variations in SRCR domain numbers (ranging from two to six), hinting at potential mechanisms influenced by disulfide bond formations.

Contrastingly, in the sea urchin, 70.47% of SRCR-SFs were organized in tandem clusters, indicating a higher prevalence of tandem gene arrangements compared to sponges. Analysis of a scaffold (NW_022145609.1) revealed a tandem array of 16 SRCR-SFs, displaying more divergent exon–intron architectures than observed in sponge SRCR-SFs. The sea urchin SRCR-SFs exhibited seven distinct exon–intron structure types, notably differing from sponge exon arrangements. Investigation into exon shuffling highlighted variations in exon–intron architectures among sibling paralogs, indicating instances of exon loss and domain modifications within SRCR-SFs.

This comparative analysis underscores the contrasting patterns of SRCR-SF distribution between sponge and sea urchin genomes. While both exhibited tandem gene arrangements as a prevalent mode of SRCR-SF expansion, differences in exon–intron structures, domain architectures, and the extent of variation within sibling paralogs highlight distinct evolutionary mechanisms driving SRCR-SF diversity in these metazoans. These findings shed light on the genetic basis of SRCR-SF expansion, contributing to a deeper understanding of evolutionary processes shaping gene families across diverse species.

### 2.7. Comparative Analysis of SRCR-SF Structures in the Sponge and Sea Urchin

**Figure 8:**
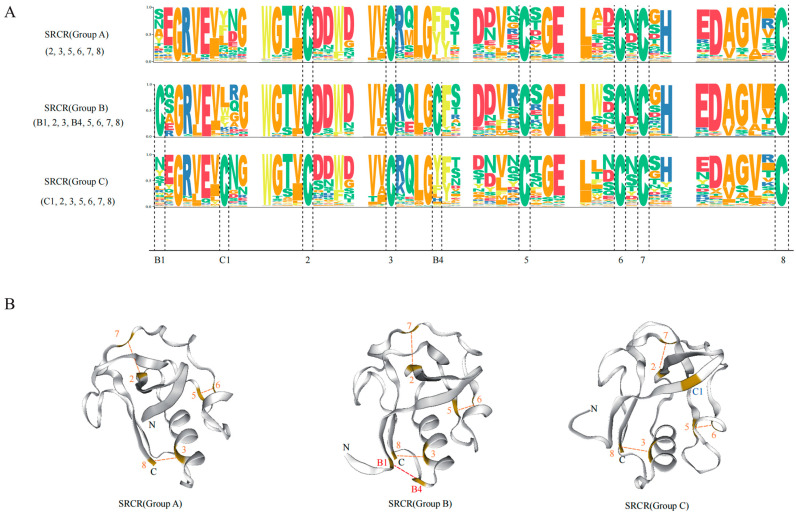
Analysis of different types of SRCR domain motifs and three-dimensional structure analysis. (**A**) Comparative analysis of motifs of Group A, Group B, and Group C SRCR domains. (**B**) Comparative analysis of the three-dimensional structures of Group A, Group B, and Group C SRCR domains. The numbers represent conserved cysteine sites.

In our investigation, motif analysis of SRCR-SFs in both the sponge and sea urchin revealed intriguing insights. SRCR domains, categorized into Group A (six cysteines) and Group B (eight cysteines) [46], unveiled an unexpected discovery: a novel type named Group C within sponge SRCR-SFs, showcasing seven conserved cysteines (Figure 8A and Supplementary Table S1). Sequence alignment indicated six shared conserved cysteines across all three groups, with the additional C1 cysteine in the novel Group C SRCR domain, distinct from the Group B SRCR domain’s cysteine conservation. This divergence suggests independent evolution of Group B with eight conserved cysteines and the newly identified Group C. Notably, genes encoding Group C SRCR domains proliferated extensively in the sponge genome, while Group A SRCR domain-rich genes expanded significantly in the sea urchin genome.

Our structural investigations, culminating in the construction of 3D models, revealed striking similarities among the predicted structures of the three SRCR domain groups (Figure 8B). Specifically, within the SRCR domain, the B1 and B4 cysteines formed an internal disulfide bond, whereas the novel C1 cysteine in the Group C SRCR domain showed no involvement in internal disulfide bonding. This observation led us to hypothesize that the novel C1 cysteine might engage in polymer formation through external disulfide bonds, signifying potential functional divergence.

To trace the evolutionary paths of these SRCR domains, particularly focusing on the novel Group C SRCR domain, we constructed a phylogenetic tree utilizing SRCR domains from six representative species spanning different evolutionary stages: *A. queenslandica*, *S. purpuratus*, *L. anatina*, *B. floridae*, *A. planci*, *C. gigas*, and *H. sapiens* (Supplementary Figure S2). Our phylogenetic analysis suggests that the Group A SRCR domain may represent an ancient architectural form. Furthermore, the prevalence of species encoding Group B SRCR domains within the vertebrate clade hints at lineage-specific duplications of SRCR-SFs harboring the Group B domain. Intriguingly, out of the 29 species examined, genes encoding Group C SRCR domains, totaling 765 genes, were predominantly identified in the sponge. Conversely, sea urchins housed only two genes, and sea stars contained just one gene encoding the Group C domain.

Additionally, to ensure high-confidence sequences, we meticulously refined the three gene models through manual optimization using transcriptomic read mapping. The presence of Group C SRCR domains across diverse clades indicates potential multiple origins or divergences, underscoring the novelty and complexity of this domain’s evolutionary history.

## 3. Discussion

Invertebrates, lacking a canonical adaptive immune system, rely entirely on their innate immune systems. Natural selection and fitness have driven the emergence of diverse survival strategies among invertebrates to thrive in pathogen-rich environments. Previous studies suggest species like the sea urchin [30] and the Pacific Oyster [31] have undergone significant immune reorganization and specificity through expanding their repertoire of innate immune receptors. However, the evolutionary history and molecular functions of SRCRs, one of the major immune receptors, remain largely unknown. In this study, we systematically delineate the evolutionary trajectory of the SRCR gene family across 29 metazoans, examining their domain architectures, phylogeny, exon–intron structures, motif patterns, and tissue-level gene expressions. To the best of our knowledge, this study represents the first comprehensive analysis of the molecular evolution of SRCR-SFs across invertebrates.

There are several exciting results we would like to highlight. Firstly, a prevalent pattern of expansion in SRCR-SFs has been observed multiple times across diverse metazoans. This expansion of SRCR-SFs in the sponge and the sea urchin coincide with similar expansions in other PRRs in several species, such as *TLRs* in the sea urchin [30], ApeC-Containing proteins in the amphioxus [47], *NLRs* in the sponge [48], and *C1qDCs* in the Pacific oyster [31]. Moreover, the degree of expansion in these PRRs varies among species, which is likely influenced by lineage-specific factors, even in the metazoan ancestor such as the sponge. These multiple time expansions underline the rapid evolution of the innate immune system in response to diverse pathogens. Our results suggest a convergent evolution of SRCR-SF expansion between sponges and sea urchins. Similar evolutionary patterns have been observed in *TLRs*, where the sea urchin genome extensively expanded V-type *TLRs*, while the Pacific oyster genome predominantly expanded short P-type *TLRs* [31]. It has been shown in the literature that expansions within gene families encoding immune recognition receptors offer a rich source of previously unrecognized immune complexity. This suggests that while invertebrate immune systems rely on similar proteins, the types of these receptors vary widely among species [49].

In our study, SRCR-SFs duplicate in the level of ‘gene’ in the sponge, while duplicating in the level of ‘domain’ in the sea urchin. Gene or domain duplications serve as primary driving forces in the evolutionary innovation of genetic systems, generating novel genes that facilitate functional divergence [50,51,52]. Tandem duplication involves structural rearrangement by serial replication and insertion of DNA segments, creating adjacent paralogous genes with short interspaces. The exon–intron structure varies among different duplicated genes under positive selection [53]. Analysis of the exon–intron structure revealed that expanded SRCR-SFs in sponges were encoded by three exons per SRCR domain, while in sea urchins, each SRCR domain was encoded by a single exon. This process is frequent among innate immune-related molecules, such as *TLRs*, *RLRs*, *NLRs*, and lectins [54]. Considering our observations regarding the expansion of SRCR-SFs in the sponge and the sea urchin, the expansion patterns of SRCR-SFs may bear similarity to those of other PRR genes. It is also worthy to note that the relationships of these expansion patterns among different gene families encoding for immune receptors require further investigation. Overall, the expansion mechanisms of immune receptor gene families are also in a lineage-specific pattern.

Secondly, molecular evolution study reveals various patterns of conserved cysteines in the sponge and sea urchin SRCR-SFs, indicating a potential different immune function role of SRCR-SFs between these species. The SRCR domain, a highly conserved component within the SRCR superfamily, encompasses over 30 proteins with diverse functions, including pathogen recognition and modulation of innate immunity. Despite this diversity, the precise roles of the SRCR domain within these proteins remain unclear [55]. Cellular mechanisms like differential splicing and post-translational modifications enable a single gene to encode numerous distinct protein products, significantly augmenting the repertoire of encoded protein functions from a finite DNA genome [56], thereby enhancing protein diversity. This adaptive process aids invertebrates in recognizing and eliminating a broad spectrum of pathogens, promoting diversity and specificity in innate immunity among invertebrates [42,49]. Similar to immunoglobulin domains, SRCR domains are multifunctional protein elements found in various biological activities and diverse domain contexts. The substantial diversity in the primary sequence of this domain potentially enables proteins to bind to a wide spectrum of ligands. In vertebrates, numerous proteins with SRCR domains play explicit immune roles by directly binding to pathogenic signatures [57]. The diverse structures and binding specificities of SRCR proteins broaden their applicability across different categories of pathogens [56,58,59,60]. This evidence also supports the idea that gene family expansions through duplication events within the genome can generate multiple protein variants or homologous proteins at the protein level.

Previous studies have highlighted the SRCR domain as the primary ligand-binding domain in MARCO [61]. This domain features conserved cysteines forming internal disulfide bonds, thereby establishing stable structures and diverse recognition regions. Notably, oxidoreductases selectively cleave disulfide bonds with relatively low reduction potentials, sparing structural bonds [62]. Hence, the formation of functional disulfide bonds critically regulates protein molecular mechanisms [63,64], which is ubiquitous across numerous proteins and pivotal in governing both their structure and function [63]. The extracellular regions of SRCR-SF members manifest either as exclusive arrays of tandemly repeated SRCR domains or as mosaic proteins comprising SRCR domains combined with various other protein modules, such as epithelial growth factor, C1r/C1s Uegf Bmp1, zona pellucida, collagenous regions, fibronectin, and short consensus repeats. Commonly observed among SRCR-SF members are short Pro, Ser, and Thr (PST)-rich polypeptides interspersed among contiguous SRCR domains. On the other hand, three-dimensional structures obtained from crystallization experiments reveal that both Group A and Group B SRCR domains exhibit a conserved and compact core folding pattern—a curved six-stranded β sheet cradling an α helix—while exhibiting variable outer loop regions, potentially contributing to functional diversity [65,66,67,68,69,70]. It is plausible to hypothesize that the cysteine content within the SRCR structure of the sponge and sea urchin, contributing to disulfide bond formation, is intricately linked to their function. These bonds likely play a role in pathogen recognition within the innate immune system, enabling these organisms to mount immune responses for self-protection. Moreover, the discovery of the Group C SRCR domain in our study might represent a novel strategy in the innate immune system’s arsenal against pathogen-induced stress. The distinct nature of this Group C SRCR domain compared to Group A and Group B SRCR domains suggests potential unique functionalities within the innate immune system. These nuances warrant further investigation for a deeper understanding of their specific roles and contributions to immune responses.

Finally, our findings indicate that numerous SRCR-SFs showed elevated expression levels within immune-related tissues, particularly the digestive glands, across nine representative metazoans with available tissue expression profiles. This suggests that the amplification of SRCR-SFs could potentially benefit specific immune functions, such as mucosal immunity, within the digestive system of these metazoans. This amplification might also play a significant role in innate immune recognition. Moreover, the question of whether proteins with identical domain architecture exhibit similar functions across different species remains unanswered [53]. Examples from the families under discussion indicate that this notion does not universally apply. For instance, while Drosophila Toll-like receptors primarily function in embryonic development, their mammalian counterparts serve as pivotal regulators of immune responses [71,72]. Therefore, the abundance of the SRCR gene family in the species of the invertebrates suggests a critical role for SRCR domains in the host defense mechanisms of those animals reliant solely on innate immunity.

## 4. Materials and Methods

### 4.1. Data Collection

In accordance with the principles of literature reporting and species representativeness, we obtained genome, protein, and annotation files for 29 metazoans from the National Center for Biotechnology Information (https://www.ncbi.nlm.nih.gov/, accessed on 1 July 2022) [73] and the OIST Marine Genomics Unit (https://marinegenomics.oist.jp/gallery, accessed on 1 July 2022), as well as other databases. All genomes used the BUSCO v5.2.2 (https://busco.ezlab.org/, accessed on 1 July 2022) suite to assess the completeness of genomes and redundancy (Supplementary Table S2). Transcriptome data of different metazoans were retrieved from the Sequence Read Archive (SRA) (https://www.ncbi.nlm.nih.gov/sra/, accessed on 1 July 2022) database on the NCBI website using the search terms “species names” and “related issues”(Supplementary Table S8).

### 4.2. Identification and Domain Annotation of SRCR-SFs

The identification of candidate SRCR-SF genes in the target gene set began with the use of the local version of HMMER 3.3.2 (http://hmmer.org/, accessed on 1 July 2022) [34]. The SRCR hmm file (PF00530 https://www.ncbi.nlm.nih.gov/Structure/cdd/PF00530, accessed on 1 July 2022) was employed, and a screening threshold of 1 × 10^−5^ was applied. Subsequently, the local version of Blastp 2.12.0+ (https://www.ncbi.nlm.nih.gov/tools/primer-blast/, accessed on 1 July 2022) [35] was utilized to screen SRCR-SF candidate genes within the target species gene set, using the SRCR seed file as input sequences, with the same screening threshold. The results from both steps were integrated, and redundant genes were removed based on information from genome annotation files. To validate the presence of SRCR-SF genes in each reference genome, the TBLASTN algorithm (https://blast.ncbi.nlm.nih.gov/, accessed on 1 July 2022) was applied using validated SRCR-SF proteins as query sequences. A significance threshold of 1 × 10^−5^ was employed for this analysis.

Following the removal of redundant SRCR-SF genes, structural batch prediction was conducted using Simple Modular Architecture Research Tool (SMART https://smart.embl.de/, accessed on 1 July 2022) [36] and the local version of InterProScan 5.55-88.0 (https://github.com/ebi-pf-team/interproscan, accessed on 1 July 2022) [37]. The Conserved Domain Database (CDD) (https://www.ncbi.nlm.nih.gov/cdd, accessed on 1 July 2022) and Pfam domain database (https://pfam.xfam.org/, accessed on 1 July 2022) were selected, and a screening threshold of 1 × 10^−5^ was applied. Refinement of selected SRCR-SF genes was performed using local GeneWise (version 2.4.1) (https://www.ebi.ac.uk/Tools/psa/genewise/, accessed on 1 July 2022) analysis and transcriptome read mapping, significantly enhancing the accuracy of each gene model. SignalP (https://services.healthtech.dtu.dk/services/SignalP-6.0/, accessed on 1 July 2022) was employed to predict signal peptides [38], while DeepTMHMM (https://dtu.biolib.com/DeepTMHMM, accessed on 1 July 2022) was utilized to predict transmembrane structures [39]. Motif analysis was carried out using Jalview (https://www.jalview.org/, accessed on 1 July 2022) to select sequences around conserved cysteines. Visualization of the motifs was performed using TBtools (https://github.com/CJ-Chen/TBtools-II, accessed on 1 July 2022) [74]. Heat maps illustrating SRCR expression in various tissues of different metazoans were generated using the pheatmap package [75] in R version 4.3.0.

### 4.3. Localization and Tandem Repeat Identification of SRCR-SF Genes

We obtained the position information of each SRCR-SF gene from the genome annotation files and visualized it through Mg2c_v2.1 (http://mg2c.iask.in/mg2c_v2.1, accessed on 1 July 2022) [76]. To determine the tandem repeat status of SRCR-SF genes, we used the following criteria: (1) adjacent genes are SRCR-SF genes; (2) the distance between two SRCR-SF genes is no more than five genes. For the tandem repeat SRCR-SF genes, we obtained the position information of exons, introns, and coding regions through genome annotation files and the results of interscan domain prediction, and we visualized them through GSDS 2.0 (http://gsds.gao-lab.org, accessed on 1 July 2022) [77].

### 4.4. SRCR Domain Structure and Phylogenetic Analysis

Obtaining the SRCR domain location information through InterProScan, the SRCR domain sequences were extracted using SeqKit 2.3.0 (https://bioinf.shenwei.me/seqkit/, accessed on 1 July 2022) [78]. Multiple sequence alignment was performed using MAFFT v7.487 (https://mafft.cbrc.jp/alignment/software/, accessed on 1 July 2022) [79] with an automatic selection of alignment strategy. The resulting alignment was trimmed using trimAl v1.4. rev15 (http://github.com/scapella/trimal, accessed on 1 July 2022) [80] with the gappyout mode. The output from MAFFT was visualized using Jalview [81] for SRCR domain type identification. The output from trimAl was used to construct a phylogenetic tree using IQ-TREE multicore v2.1.4-beta (http://www.iqtree.org, accessed on 1 July 2022) [82] with a maximum likelihood method, automatic model selection, fast bootstrap with 1000 replicates, and SH-like approximate likelihood ratio test with 1000 replicates to calculate node support. The resulting tree file was visualized using iTOL v5 (https://itol.embl.de, accessed on 1 July 2022) [83].

## 5. Conclusions

In this study, we systematically explored the evolutionary dynamics of SRCR-SFs in the metazoans, employing an integrated framework encompassing comparative genomics, structural domain architecture, exon–intron structure, phylogenetic insights, and tissue expression profiles. Our exhaustive investigation led to the identification of a large number of genes coding for SRCR-SFs in metazoans, notably 296 in the sponge and 357 in the sea urchin. This amplification might play a significant role in the diversity and specificity of innate immune recognition. Phylogenetic analysis revealed the expansion of SRCR-SFs within sponge and sea urchin strains, elucidating their species-specific and independent evolutionary paths. In addition, cysteine-rich SRCR domains in the sponge and sea urchin contribute to the formation of disulfide bonds that may play a key role in their innate immune response. The discovery of SRCR domains in Group C not only provides a potential strategy for defense against pathogen-induced stress, but also suggests a unique function of the innate immune system. Finally, the amplified presence of SRCR-SFs, especially in sponge and sea urchin lineages, correlates with heightened expression levels in specific metazoan tissues, notably those associated with digestive functions. These results are expected to shed light on the complex innate immune system that the inveterate utilized as alternative strategies to recognize various pathogens.

## Figures and Tables

**Table 1:** The top five domain compositions covering more than 50% of the SRCR-SFs of the 29 representative species grouped by clusters.

Species	*A. queenslandica*	*P. australis*	*C. gigas*	*S. purpuratus*	*A. planci*	*D. rerio*	*G. gallus*
	EGF_CA (PF07645)	CUB (PF00431)	TSP 1 (PF00090)	EGF-like (PF00008)	Sushi repeat (PF00084)	Trypsin (PF00089)	Trypsin (PF00089)
	cEGF (PF12662)	Kringle (PF00051)	LDL A (PF00057)	Sushi repeat (PF00084)	EGF-like (PF00008)	Collagen (PF01391)	LDL-A (PF00057)
Domain types	FXa_inhibition (PF14670)	WSC (PF01822)	EGF-like (PF00008)	F5/8 type C (PF00754)	LDL-A (PF00057)	Ig_3 (PF13927)	Collagen (PF01391)
	PK_Tyr_Ser-Thr (PF07714)	LDL-A (PF00057)	MAM (PF00629)	WSC (PF01822)	F5/8 type C (PF00754)	Lysyl oxidase (PF01186)	CUB (PF00431)
	Astacin (PF01400)	PKD (PF00801)	EGF_CA (PF07645)	CUB (PF00431)	7tm_2 (PF00002)	Kringle (PF00051)	Lysyl oxidase (PF01186)

## Data Availability

Data is contained within the article.

## Supplementary Materials

The supporting information can be downloaded at: https://www.mdpi.com/article/10.3390/ijms25031515/s1.

## References

[1] Pechinskii S.V., Kuregyan A.G. (2014). The Impact of Carotenoids on Immunity. Pharm. Chem. J..

[2] Hibino T., Loza-Coll M., Messier C., Majeske A.J., Cohen A.H., Terwilliger D.P., Buckley K.M., Brockton V., Nair S.V., Berney K. (2006). The immune gene repertoire encoded in the purple sea urchin genome. Dev. Biol..

[3] Peiris T.H., Hoyer K.K., Oviedo N.J. (2014). Innate immune system and tissue regeneration in planarians: An area ripe for exploration. Semin. Immunol..

[4] Sousa H., Hinzmann M. (2020). Review: Antibacterial components of the Bivalve’s immune system and the potential of freshwater bivalves as a source of new antibacterial compounds. Fish Shellfish Immunol..

[5] Kurtz J. (2004). Memory in the innate and adaptive immune systems. Microbes Infect..

[6] Murphy K., Weaver C. (2016). Janeway’s Immunobiology.

[7] Prigot-Maurice C., Beltran-Bech S., Braquart-Varnier C. (2022). Why and how do protective symbionts impact immune priming with pathogens in invertebrates?. Dev. Comp. Immunol..

[8] Verma M., Michalec L., Sripada A., McKay J., Sirohi K., Dyjack N.T., Gorska M.M., Seibold M., Martin R.J., Alam R. (2019). Innate Lymphoid Cells (ILCs) Generate Memory for Pathogen-Associated Molecular Patterns (PAMPs) of Allergens, Which Contributes to Asthma. J. Allergy Clin. Immunol..

[9] Akira S., Uematsu S., Takeuchi O. (2006). Pathogen Recognition and Innate Immunity. Cell.

[10] Shekarian T., Valsesia-Wittmann S., Brody J., Michallet M.C., Depil S., Caux C., Marabelle A. (2017). Pattern recognition receptors: Immune targets to enhance cancer immunotherapy. Ann. Oncol..

[11] Zhao Q., Wang Q., Wang T., Xu J., Li T., Liu Q., Yao Q., Wang P. (2021). Pattern Recognition Receptors (PRRs) in Macrophages Possess Prognosis and Immunotherapy Potential for Melanoma. Front. Immunol..

[12] Taghavi M., Khosravi A., Mortaz E., Nikaein D., Athari S.S. (2017). Role of pathogen-associated molecular patterns (PAMPS) in immune responses to fungal infections. Eur. J. Pharmacol..

[13] Janeway C.A. (1989). Approaching the asymptote? Evolution and revolution in immunology. Cold Spring Harb. Symp. Quant. Biol..

[14] Freeman M., Ashkenas J., Rees D.J., Kingsley D.M., Copeland N.G., Jenkins N.A., Krieger M. (1990). An ancient, highly conserved family of cysteine-rich protein domains revealed by cloning type I and type II murine macrophage scavenger receptors. Proc. Natl. Acad. Sci. USA.

[15] Bowdish D.M., Gordon S. (2009). Conserved domains of the class A scavenger receptors: Evolution and function. Immunol. Rev..

[16] Kang W., Nielsen O., Fenger C., Madsen J., Hansen S., Tornoe I., Eggleton P., Reid K.B., Holmskov U. (2002). The scavenger receptor, cysteine-rich domain-containing molecule gp-340 is differentially regulated in epithelial cell lines by phorbol ester. Clin. Exp. Immunol..

[17] Hohenester E., Sasaki T., Timpl R. (1999). Crystal structure of a scavenger receptor cysteine-rich domain sheds light on an ancient superfamily. Nat. Struct. Biol..

[18] Sarrias M.R., Grønlund J., Padilla O., Madsen J., Holmskov U., Lozano F. (2004). The Scavenger Receptor Cysteine-Rich (SRCR) domain: An ancient and highly conserved protein module of the innate immune system. Crit. Rev. Immunol..

[19] Casadó-Llombart S., Velasco-de Andrés M., Català C., Leyton-Pereira A., Gutiérrez-Cózar R., Suárez B., Armiger N., Carreras E., Esteller M., Ricart E. (2022). Experimental and genetic evidence for the impact of CD5 and CD6 expression and variation in inflammatory bowel disease. Front. Immunol..

[20] Qiu R., Sun B.-G., Li J., Liu X., Sun L. (2013). Identification and characterization of a cell surface scavenger receptor cysteine-rich protein of Sciaenops ocellatus: Bacterial interaction and its dependence on the conserved structural features of the SRCR domain. Fish Shellfish Immunol..

[21] Reichhardt M.P., Loimaranta V., Lea S.M., Johnson S. (2020). Structures of SALSA/DMBT1 SRCR domains reveal the conserved ligand-binding mechanism of the ancient SRCR fold. Life Sci. Alliance.

[22] Kanno S., Hirano S., Sakamoto T., Furuyama A., Takase H., Kato H., Fukuta M., Aoki Y. (2020). Scavenger receptor MARCO contributes to cellular internalization of exosomes by dynamin-dependent endocytosis and macropinocytosis. Sci. Rep..

[23] Whitley K.V., Freitas C.M.T., Moreno C., Haynie C., Bennett J., Hancock J.C., Cox T.D., Pickett B.E., Weber K.S. (2022). CD5 Deficiency Alters Helper T Cell Metabolic Function and Shifts the Systemic Metabolome. Biomedicines.

[24] Ma L., Huang C., Wang X.J., Xin D.E., Wang L.S., Zou Q.C., Zhang Y.S., Tan M.D., Wang Y.M., Zhao T.C. (2017). Lysyl Oxidase 3 Is a Dual-Specificity Enzyme Involved in STAT3 Deacetylation and Deacetylimination Modulation. Mol. Cell.

[25] Pahler S., Blumbach B., Müller I., Müller W.E.G. (1998). Putative multiadhesive protein from the marine sponge Geodia cydonium: Cloning of the cDNA encoding a fibronectin-, an SRCR-, and a complement control protein module. J. Exp. Zool..

[26] Gonçalves C.M., Castro M.A.A., Henriques T., Oliveira M.I., Pinheiro H.C., Oliveira C., Sreenu V.B., Evans E.J., Davis S.J., Moreira A. (2009). Molecular cloning and analysis of SSc5D, a new member of the scavenger receptor cysteine-rich superfamily. Mol. Immunol..

[27] Solé R. (2022). Revisiting Leigh Van Valen’s “A New Evolutionary Law” (1973). Biol. Theory.

[28] Martínez V.G., Moestrup S.K., Holmskov U., Mollenhauer J., Lozano F. (2011). The conserved scavenger receptor cysteine-rich superfamily in therapy and diagnosis. Pharmacol. Rev..

[29] Sarukhan A., Martinez-Florensa M., Escoda-Ferran C., Carrasco E., Carreras E., Lozano F. (2016). Pattern Recognition by CD6: A Scavenger-Like Lymphocyte Receptor. Curr. Drug Targets.

[30] Rast J.P., Smith L.C., Loza-Coll M., Hibino T., Litman G.W. (2006). Genomic Insights into the Immune System of the Sea Urchin. Science.

[31] Zhang L., Li L., Guo X., Litman G.W., Dishaw L.J., Zhang G. (2015). Massive expansion and functional divergence of innate immune genes in a protostome. Sci. Rep..

[32] Huang S., Yuan S., Guo L., Yu Y., Li J., Wu T., Liu T., Yang M., Wu K., Liu H. (2008). Genomic analysis of the immune gene repertoire of amphioxus reveals extraordinary innate complexity and diversity. Genome Res..

[33] Liu L., Yang J., Qiu L., Wang L., Zhang H., Wang M., Vinu S.S., Song L. (2011). A novel scavenger receptor-cysteine-rich (SRCR) domain containing scavenger receptor identified from mollusk mediated PAMP recognition and binding. Dev. Comp. Immunol..

[34] Wheeler T.J., Eddy S.R. (2013). nhmmer: DNA homology search with profile HMMs. Bioinformatics.

[35] Ye J., Coulouris G., Zaretskaya I., Cutcutache I., Rozen S., Madden T.L. (2012). Primer-BLAST: A tool to design target-specific primers for polymerase chain reaction. BMC Bioinform..

[36] Letunic I., Bork P. (2017). 20 years of the SMART protein domain annotation resource. Nucleic Acids Res..

[37] Jones P., Binns D., Chang H.Y., Fraser M., Li W., McAnulla C., McWilliam H., Maslen J., Mitchell A., Nuka G. (2014). InterProScan 5: Genome-scale protein function classification. Bioinformatics.

[38] Teufel F., Almagro Armenteros J.J., Johansen A.R., Gíslason M.H., Pihl S.I., Tsirigos K.D., Winther O., Brunak S., von Heijne G., Nielsen H. (2022). SignalP 6.0 predicts all five types of signal peptides using protein language models. Nat. Biotechnol..

[39] Jeppe H., Konstantinos D.T., Mads Damgaard P., José Juan Almagro A., Paolo M., Henrik N., Anders K., Ole W. (2022). DeepTMHMM predicts alpha and beta transmembrane proteins using deep neural networks. bioRxiv.

[40] Harris R.M., Hofmann H.A. (2015). Seeing is believing: Dynamic evolution of gene families. Proc. Natl. Acad. Sci. USA.

[41] Cho S.-J., Vallès Y., Giani V.C., Seaver E.C., Weisblat D.A. (2010). Evolutionary Dynamics of the wnt Gene Family: A Lophotrochozoan Perspective. Mol. Biol. Evol..

[42] Li Y., Xue Y., Peng Z., Zhang L. (2023). Immune diversity in lophotrochozoans, with a focus on recognition and effector systems. Comput. Struct. Biotechnol. J..

[43] Canciani A., Catucci G., Forneris F. (2019). Structural characterization of the third scavenger receptor cysteine-rich domain of murine neurotrypsin. Protein Sci..

[44] Zhu Y., Wu N., Song W., Yin G., Qin Y., Yan Y., Hu Y. (2014). Soybean (Glycine max) expansin gene superfamily origins: Segmental and tandem duplication events followed by divergent selection among subfamilies. BMC Plant Biol..

[45] Huang S., Chen Z., Yan X., Yu T., Huang G., Yan Q., Pontarotti P.A., Zhao H., Li J., Yang P. (2014). Decelerated genome evolution in modern vertebrates revealed by analysis of multiple lancelet genomes. Nat. Commun..

[46] Resnick D., Chatterton J.E., Schwartz K., Slayter H., Krieger M. (1996). Structures of class A macrophage scavenger receptors. Electron microscopic study of flexible, multidomain, fibrous proteins and determination of the disulfide bond pattern of the scavenger receptor cysteine-rich domain. J. Biol. Chem..

[47] Li Y., Li J., Yan X., Chen S., Wu C., Huang H., Shi Y., Huang G., Dong M., Xu A. (2021). Broad distribution, high diversity and ancient origin of the ApeC-containing proteins. Mol. Phylogenet. Evol..

[48] Yuen B., Bayes J.M., Degnan S.M. (2013). The Characterization of Sponge NLRs Provides Insight into the Origin and Evolution of This Innate Immune Gene Family in Animals. Mol. Biol. Evol..

[49] Buckley K.M., Rast J.P. (2015). Diversity of animal immune receptors and the origins of recognition complexity in the deuterostomes. Dev. Comp. Immunol..

[50] Vogel C., Teichmann S.A., Pereira-Leal J. (2005). The relationship between domain duplication and recombination. J. Mol. Biol..

[51] de Souza S.J. (2012). Domain shuffling and the increasing complexity of biological networks. Bioessays.

[52] Magadum S., Banerjee U., Murugan P., Gangapur D., Ravikesavan R. (2013). Gene duplication as a major force in evolution. J. Genet..

[53] Yao S., Chan J., Xu Y., Wu S., Zhang L. (2022). Divergences of the RLR Gene Families across Lophotrochozoans: Domain Grafting, Exon-Intron Structure, Expression, and Positive Selection. Int. J. Mol. Sci..

[54] Oren M., Barela Hudgell M.A., D’Allura B., Agronin J., Gross A., Podini D., Smith L.C. (2016). Short tandem repeats, segmental duplications, gene deletion, and genomic instability in a rapidly diversified immune gene family. BMC Genom..

[55] Huang F.-L., Shiao Y.-J., Hou S.-J., Yang C.-N., Chen Y.-J., Lin C.-H., Shie F.-S., Tsay H.-J. (2013). Cysteine-rich domain of scavenger receptor AI modulates the efficacy of surface targeting and mediates oligomeric Aβ internalization. J. Biomed. Sci..

[56] Boulanger L.M. (2009). Immune proteins in brain development and synaptic plasticity. Neuron.

[57] End C., Bikker F., Renner M., Bergmann G., Lyer S., Blaich S., Hudler M., Helmke B., Gassler N., Autschbach F. (2009). DMBT1 functions as pattern-recognition molecule for poly-sulfated and poly-phosphorylated ligands. Eur. J. Immunol..

[58] Iwasaki A., Medzhitov R. (2015). Control of adaptive immunity by the innate immune system. Nat. Immunol..

[59] Pees B., Yang W., Zárate-Potes A., Schulenburg H., Dierking K. (2015). High Innate Immune Specificity through Diversified C-Type Lectin-Like Domain Proteins in Invertebrates. J. Innate Immun..

[60] Turvey S.E., Broide D.H. (2010). Innate immunity. J. Allergy Clin. Immunol..

[61] Chen Y., Sankala M., Ojala J.R., Sun Y., Tuuttila A., Isenman D.E., Tryggvason K., Pikkarainen T. (2006). A phage display screen and binding studies with acetylated low density lipoprotein provide evidence for the importance of the scavenger receptor cysteine-rich (SRCR) domain in the ligand-binding function of MARCO. J. Biol. Chem..

[62] Hogg P.J. (2009). Contribution of allosteric disulfide bonds to regulation of hemostasis. J. Thromb. Haemost..

[63] Pijning A.E., Hogg P. (2019). Classification of Protein Disulphide Bonds. Methods Mol. Biol..

[64] Butera D., Passam F., Ju L., Cook K.M., Woon H., Aponte-Santamaría C., Gardiner E., Davis A.K., Murphy D.A., Bronowska A. (2018). Autoregulation of von Willebrand factor function by a disulfide bond switch. Sci. Adv..

[65] Ojala J.R., Pikkarainen T., Tuuttila A., Sandalova T., Tryggvason K. (2007). Crystal structure of the cysteine-rich domain of scavenger receptor MARCO reveals the presence of a basic and an acidic cluster that both contribute to ligand recognition. J. Biol. Chem..

[66] Rodamilans B., Muñoz I.G., Bragado-Nilsson E., Sarrias M.R., Padilla O., Blanco F.J., Lozano F., Montoya G. (2007). Crystal structure of the third extracellular domain of CD5 reveals the fold of a group B scavenger cysteine-rich receptor domain. J. Biol. Chem..

[67] Timmerman L.A., Clipstone N.A., Ho S.N., Northrop J.P., Crabtree G.R. (1996). Rapid shuttling of NF-AT in discrimination of Ca^2+^ signals and immunosuppression. Nature.

[68] Garza-Garcia A., Esposito D., Rieping W., Harris R., Briggs C., Brown M.H., Driscoll P.C. (2008). Three-dimensional solution structure and conformational plasticity of the N-terminal scavenger receptor cysteine-rich domain of human CD5. J. Mol. Biol..

[69] Telfer J.C., Hsu H., Tyner M.D., Le Page L. (2022). Assessment of Scavenger Receptor Cysteine-Rich Domain Binding to Bacteria. Methods Mol. Biol..

[70] Somoza J.R., Ho J.D., Luong C., Ghate M., Sprengeler P.A., Mortara K., Shrader W.D., Sperandio D., Chan H., McGrath M.E. (2003). The structure of the extracellular region of human hepsin reveals a serine protease domain and a novel scavenger receptor cysteine-rich (SRCR) domain. Structure.

[71] Zambounis A., Elias M., Sterck L., Maumus F., Gachon C.M.M. (2011). Highly Dynamic Exon Shuffling in Candidate Pathogen Receptors…What if Brown Algae Were Capable of Adaptive Immunity?. Mol. Biol. Evol..

[72] Kambris Z., Hoffmann J.A., Imler J.L., Capovilla M. (2002). Tissue and stage-specific expression of the Tolls in Drosophila embryos. Gene Expr. Patterns.

[73] Sayers E.W., Beck J., Bolton E.E., Bourexis D., Brister J.R., Canese K., Comeau D.C., Funk K., Kim S., Klimke W. (2021). Database resources of the National Center for Biotechnology Information. Nucleic Acids Res..

[74] Chen C., Chen H., Zhang Y., Thomas H.R., Frank M.H., He Y., Xia R. (2020). TBtools: An Integrative Toolkit Developed for Interactive Analyses of Big Biological Data. Mol. Plant.

[75] Kolde R., Kolde M.R. (2015). Package ‘pheatmap’. R Package.

[76] Chao J., Li Z., Sun Y., Aluko O.O., Wu X., Wang Q., Liu G. (2021). MG2C: A user-friendly online tool for drawing genetic maps. Mol. Hortic..

[77] Hu B., Jin J., Guo A.-Y., Zhang H., Luo J., Gao G. (2014). GSDS 2.0: An upgraded gene feature visualization server. Bioinformatics.

[78] Shen W., Le S., Li Y., Hu F. (2016). SeqKit: A Cross-Platform and Ultrafast Toolkit for FASTA/Q File Manipulation. PLoS ONE.

[79] Katoh K., Rozewicki J., Yamada K.D. (2019). MAFFT online service: Multiple sequence alignment, interactive sequence choice and visualization. Brief. Bioinform..

[80] Capella-Gutiérrez S., Silla-Martínez J.M., Gabaldón T. (2009). trimAl: A tool for automated alignment trimming in large-scale phylogenetic analyses. Bioinformatics.

[81] Waterhouse A.M., Procter J.B., Martin D.M., Clamp M., Barton G.J. (2009). Jalview Version 2—A multiple sequence alignment editor and analysis workbench. Bioinformatics.

[82] Minh B.Q., Schmidt H.A., Chernomor O., Schrempf D., Woodhams M.D., von Haeseler A., Lanfear R. (2020). IQ-TREE 2: New Models and Efficient Methods for Phylogenetic Inference in the Genomic Era. Mol. Biol. Evol..

[83] Letunic I., Bork P. (2021). Interactive Tree of Life (iTOL) v5: An online tool for phylogenetic tree display and annotation. Nucleic Acids Res..

