# Supplementary material for: Genome-Wide Comparative Analysis of SRCR Gene Superfamily in Invertebrates Reveals Massive and Independent Gene Expansions in the Sponge and Sea Urchin
Source: Int J Mol Sci. 2024 Jan 26;25(3):1515. doi: 10.3390/ijms25031515 (PMC10855680; doi:10.3390/ijms25031515)
Supplement: Supplementary file 1 [file ijms-25-01515-s001.zip › Supplementary Material Description.pdf]

Supplementary Material Description

Figure S1. This figure displays the phylogenetic analysis of the SRCR domains contained in the genes shown in Figure 3. LOCXXX represents the gene name, and X in SRCRX indicates the position of the SRCR domain. The study indicates that SRCR domains located at odd positions cluster together, and those at even positions cluster together, further supporting the hypothesis that tandem duplication of SRCR-SFs in sponges occurs with a "unit" of SRCR-SRCR.

Figure S2. Evolutionary analysis of different types of SRCR domains. Different colors represent different species and different types of domains. This evolutionary tree includes 7 animals, namely *A. queenslandica, H. sapiens, S. purpuratus, A planci, B. floridae, C. gigas, and L. anatina.*

Figure S3. This figure illustrates the distribution of the number of tandem and scatter groups in the clusters of SRCR-SFs identified in 29 representative species

Table S1. Statistical analysis of SRCR domain types (Including complete and incomplete types). Structures with 6, 7, and 8 cysteine residues are considered as standard complete SRCR domains, while SRCR domains with other numbers of cysteine residues are considered as incomplete types.

Table S2. Data sources for genomes, protein sets and annotated files of 29 representative species. This table lists the taxonomic information, species names, contigN50, and data source links involved in this study.

Table S3. Identification of clusters for SRCR-SFs in 29 representative species.

Table S4. The structural domain composition pattern types of the SRCR gene family based on raw data. This table is a supplement to Figure 1, which counts the number of different domain combination types for each species.

Table S5. Statistical analysis of the structural domain composition of the SRCR gene family. The SRCR genes identified in 29 species are composed of 125 domains arranged in different numbers and ways. This table counts the frequency of each domain used in each species.

Table S6. InterProScan annotation results for members of the SRCR gene family. This table shows the annotation results of the identified SRCR genes using Interproscan, and each domain contained in each SRCR gene is annotated (using the PFAM database).

Table S7. Gene ID corresponding to the annotation file of the custom ID. For convenience of analysis, we renamed the gene names of the species. This table lists the correspondence between the gene names used in our analysis and the gene names in the database.

Table S8. Information on data sources used for analyzing the expression of tandem duplicated genes in different species and tissues.
