# Supplementary figures and images for: Genome-Wide Comparative Analysis of SRCR Gene Superfamily in Invertebrates Reveals Massive and Independent Gene Expansions in the Sponge and Sea Urchin
Source: Int J Mol Sci. 2024 Jan 26;25(3):1515. doi: 10.3390/ijms25031515 (PMC10855680; doi:10.3390/ijms25031515)

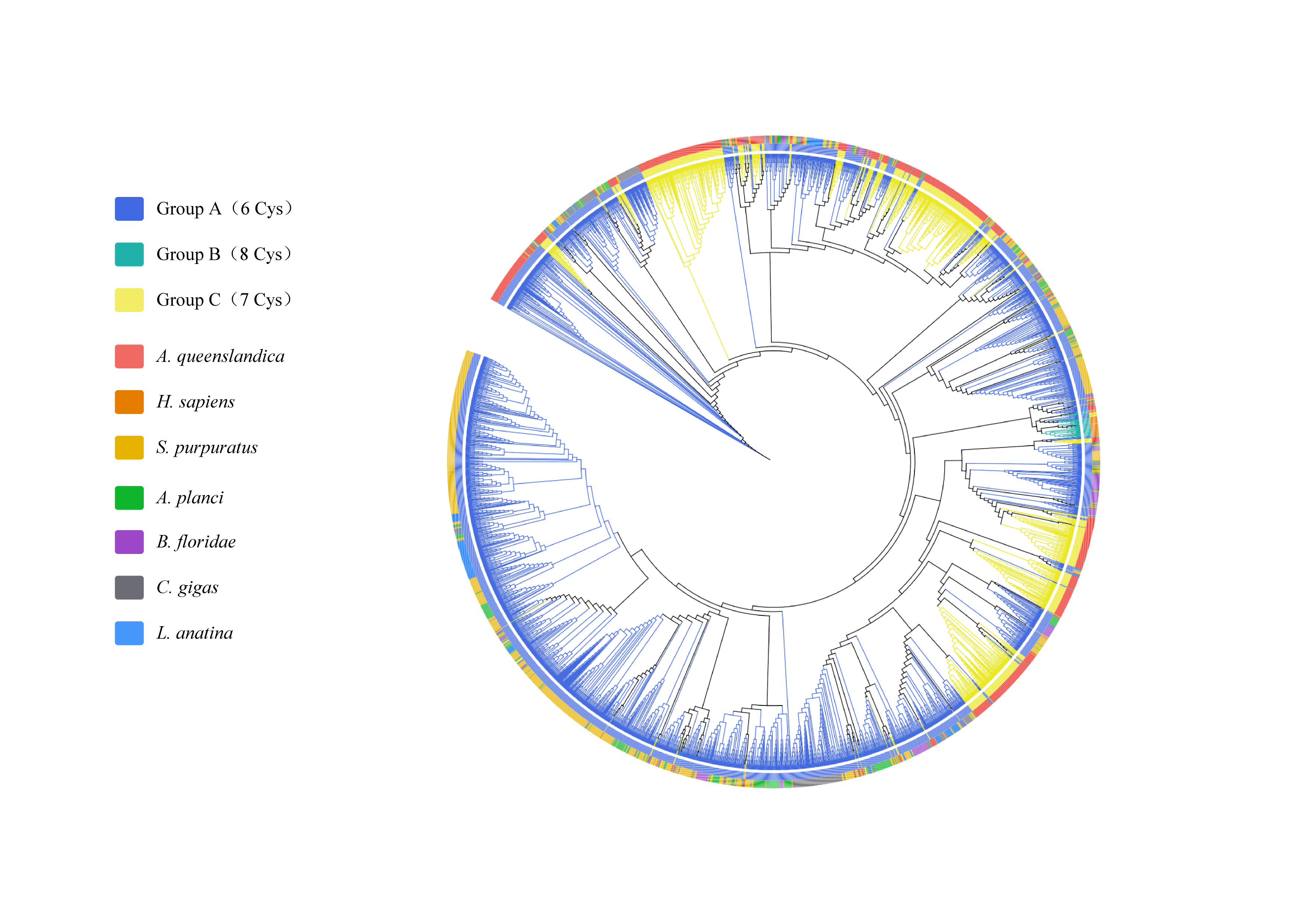

Supplement: Supplementary file 1 [file ijms-25-01515-s001.zip › Figure S2.tif]

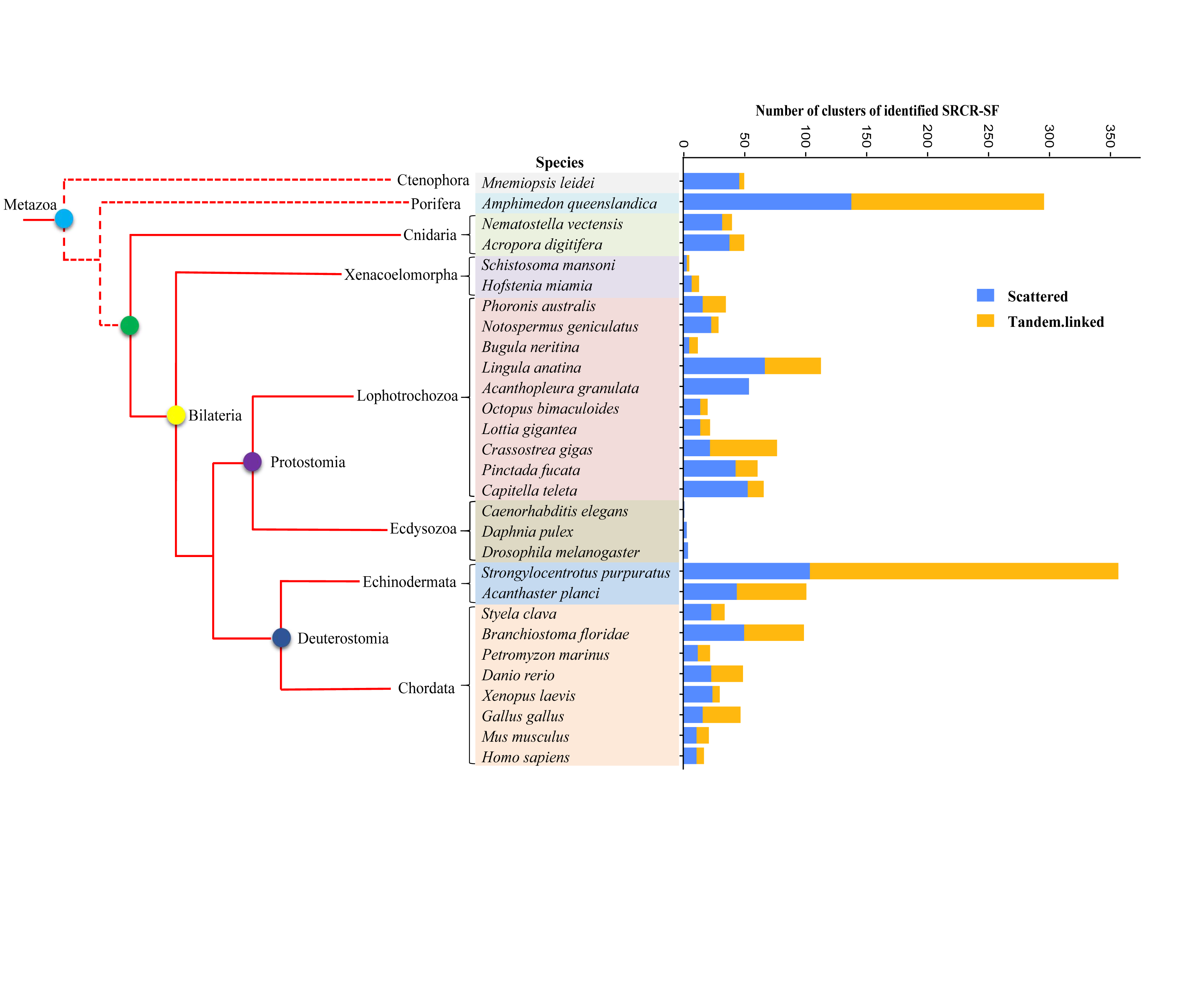

Supplement: Supplementary file 1 [file ijms-25-01515-s001.zip › Figure S3.tif]
